# Characteristics of Adaptation in Undergraduate University Students Suddenly Exposed to Fully Online Education During the COVID-19 Pandemic

**DOI:** 10.3389/fpsyt.2021.731137

**Published:** 2021-09-13

**Authors:** Daiki Ishimaru, Hiroyoshi Adachi, Hajime Nagahara, Shizuka Shirai, Haruo Takemura, Noriko Takemura, Alizadeh Mehrasa, Teruo Higashino, Yasushi Yagi, Manabu Ikeda

**Affiliations:** ^1^Department of Psychiatry, Osaka University Graduate School of Medicine, Suita, Japan; ^2^Health and Counseling Center, Osaka University, Toyonaka, Japan; ^3^Department of Artificial Intelligence and Mathematics, Institute for Datability Science, Osaka University, Suita, Japan; ^4^Infomedia Education Research Division, Cybermedia Center, Osaka University, Toyonaka, Japan; ^5^Language Education Support Research Division, Cybermedia Center, Osaka University, Toyonaka, Japan; ^6^Department of Information Networking, Osaka University Graduate School of Information Science and Technology, Suita, Japan; ^7^Department of Intelligent Media, The Institute of Scientific and Industrial Research, Osaka University, Ibaraki, Japan

**Keywords:** school adaptation, mental health, online learning, University students, COVID-19

## Abstract

This study aimed to clarify the adaptation features of University students exposed to fully online education during the novel coronavirus disease 2019 (COVID-19) pandemic and to identify accompanying mental health problems and predictors of school adaptation. The pandemic has forced many universities to transition rapidly to delivering online education. However, little is known about the impact of this drastic change on students' school adaptation. This cross-sectional study used an online questionnaire, including assessments of impressions of online education, study engagement, mental health, and lifestyle habits. In total, 1,259 students were assessed. The characteristics of school adaptation were analyzed by a two-step cluster analysis. The proportion of mental health problems was compared among different groups based on a cluster analysis. A logistic regression analysis was used to identify predictors of cluster membership. *P*-values < 0.05 were considered statistically significant. The two-step cluster analysis determined three clusters: school adaptation group, school maladaptation group, and school over-adaptation group. The last group significantly exhibited the most mental health problems. Membership of this group was significantly associated with being female (OR = 1.42; 95% CI 1.06–1.91), being older (OR = 1.21; 95% CI 1.01–1.44), those who considered online education to be less beneficial (OR = 2.17; 95% CI 1.64–2.88), shorter sleep time on weekdays (OR = 0.826; 95% CI 0.683–.998), longer sleep time on holidays (OR = 1.21; 95% CI 1.03–1.43), and worse restorative sleep (OR = 2.27; 95% CI 1.81–2.86). The results suggest that academic staff should understand distinctive features of school adaptation owing to the rapid transition of the educational system and should develop support systems to improve students' mental health. They should consider ways to incorporate online classes with their lectures to improve students' perceived benefits of online education. Additionally, educational guidance on lifestyle, such as sleep hygiene, may be necessary.

## Introduction

With the onset of the novel coronavirus disease 2019 (COVID-19) pandemic, in 2020 (1), several countries adopted emergency measures, such as quarantines, restrictions on movement, and urban lockdowns to prevent the spread of infection. In Japan, a state of emergency was declared on April 7, 2020, requesting people to refrain from leaving their homes. Resultantly, many people have had to change their lifestyle (2), affecting work, leisure, and student life. Most universities have rapidly transitioned their educational programs from traditional face-to-face teaching to online delivery modes (3–5). The majority of University students in Japan were suddenly exposed to a fully online education system. Prior to the pandemic, several studies sought to investigate the advantages and disadvantages of online education, while integrating them into traditional classes (6, 7). However, long-term and large-scale surveys, in which many students experience an accumulative burden of physical and mental conditions, are very difficult to implement because of ethical considerations. Coincidentally, changes in the educational system with the onset of the COVID-19 pandemic provided the first opportunity to examine the effect of applying a fully online education system for a long period on many University students.

On the one hand, the literature has revealed the following advantages of online education during the pandemic: remote learning, accessibility of programs, and asynchronous learning (8). On the other hand, there are also several disadvantages. For example, some studies identified the following challenges of online courses: technological problems related to communication, student assessment, use of technology tools, and uncertainty about assessments or exams (4, 9). Although the various impacts of online education on students have been attracting attention in recent years, very few studies have been conducted to evaluate students' adaptation to online education. As a crucial issue regarding school adaptation, many previous studies have reported increased mental health problems, such as anxiety, depression, or stress, among University students during the COVID-19 pandemic (10–12), while identifying several associated factors such as demographic features or lifestyle habits including diet and sleep. In contrast, to the best of our knowledge, limited data exist to investigate the mental health features of University students during the COVID-19 pandemic in the context of adaptation to online education. Given that the social isolation measures during the COVID-19 pandemic, as with lockdowns or quarantines, may cause mental health distress (13, 14), a fully online education system potentially increases the likelihood of students developing mental health problems.

This study aimed to clarify the features of school adaptation in University students exposed to a fully online education system during the COVID-19 pandemic and to identify the accompanying mental health problems and predictors of school adaptation.

## Method

### Participants

This study was conducted at Osaka University in Osaka Prefecture, Japan, from July 27, 2020 to August 10, 2020. Participants were regular affiliation students in their first year of undergraduate study who were currently enrolled at Osaka University, excluding those temporarily absent or studying abroad. In the Japanese education system, University students, with the exception of first year undergraduates, have few opportunities to attend common classes due to faculty-specific experiments or academic activities. As such, this study focuses on just the first year of undergraduate study to reveal the impact of sudden exposure to a fully online education system. Inclusion criteria were enrolment in all online-type lectures, which consisted of real-time classes, on-demand classes, and lecture material distribution classes. Students were excluded if they had any missing assessment data.

### Procedure

This study was a part of the survey which sought to investigate the content and feature of online education provided to University students in the Osaka University during the COVID-19 pandemic, while also focusing on the characteristics of school adaptation and mental health for them. This study used a cross-sectional and observational design. The survey used an online questionnaire for all potential participants to assess their attendance status for online classes and mental health parameters. The study tool was distributed among participants by the online education support system of Osaka University and accessed by them using their own PC, tablet, or smartphone. In the online education support system, information about all students in Osaka University was registered. Students could not make multiple identifiers to answer the questionnaire. The questionnaire included a written explanation that completing the questionnaire implied informed consent; participation in the study was voluntary; and no negative consequences would occur if they chose not to participate.

**Figure 1:**
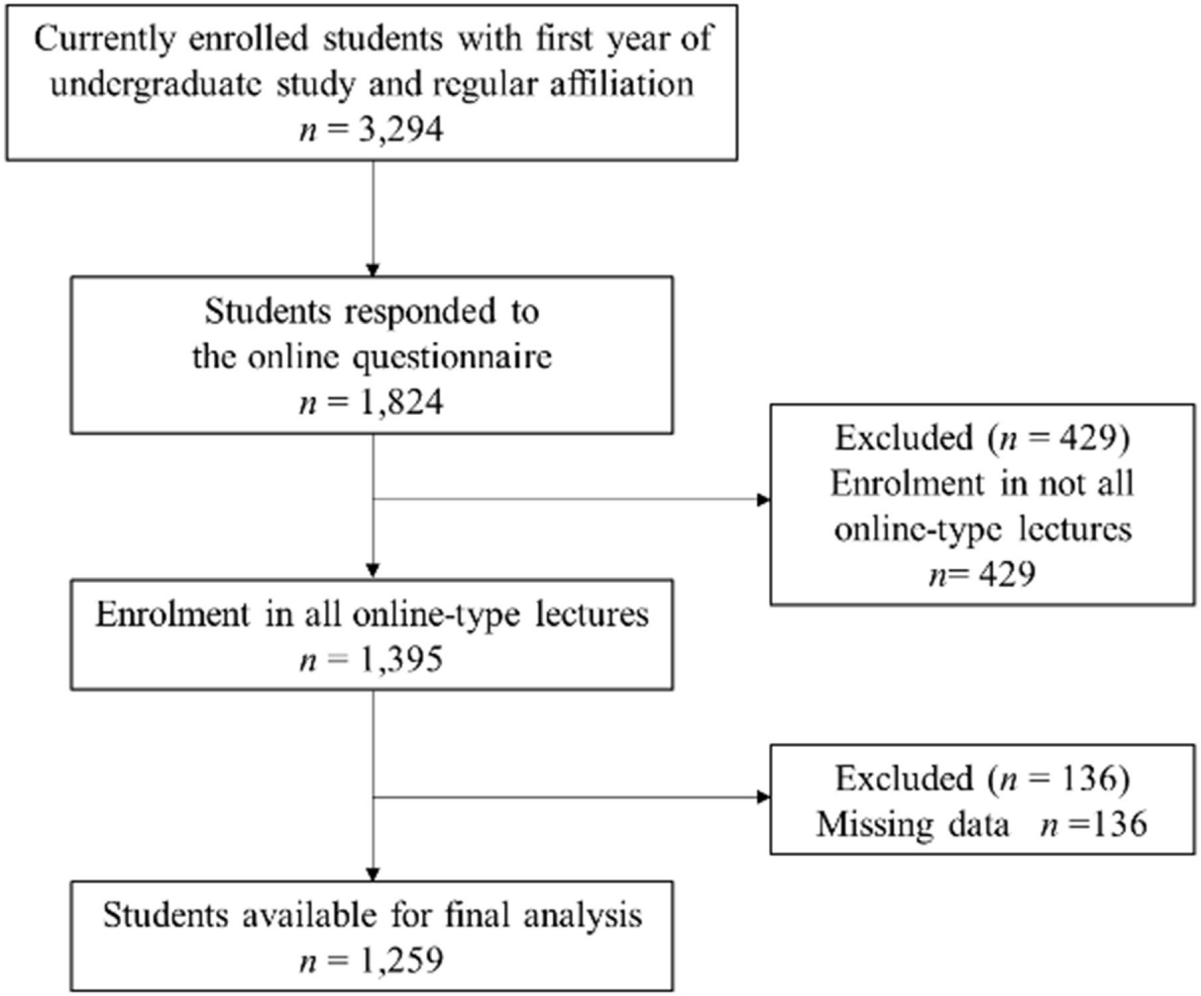
Flow diagram of participants who met inclusion/exclusion criteria.

In total, 15,194 undergraduate students were registered at Osaka University. First year undergraduate students comprised 22.4% of the total number of students. The online questionnaire was sent to 3,294 students; 1,824 students responded (55.4%). Based on the eligibility criteria, 1,259 students (38.2%) were included in the analysis (Figure 1).

### Measures

The questionnaire employed in this study is presented in the Supplementary File. Further details of the measures are presented below under the respective variable subsections.

### Demographics

The demographic data of the participants were obtained via the online education support system of Osaka University. The variables included their sex and age. The faculty of the respective classes that participants attended were also assessed since it was postulated that the degree or content of students' experience with online classes were likely to vary depending on the faculty member.

### Impression of Online Education

Participants were asked to evaluate the level of subjective benefits of online education on a scale of 1 to 5 (questionnaire presented as item 6 in the Supplementary File); lower ratings indicated that online education had more advantages than face-to-face education. Participant responses were classified into yes (score of 1 and 2), no (score of 4 and 5), or not applicable (score of 3).

### Study Engagement

The Japanese version of the Utrecht Work Engagement Scale for Students (UWES-S-J) was used to assess study engagement (15). It consists of 14 items with three sub-domains: vigor, dedication, and absorption. All items were rated on a 7-point scale (0 = never; 6 = always). Total scores ranged from 0 to 84; higher scores indicated better study engagement. The terms of each question were modified for assessing engagement with online education.

The UWES-S-J has been reported to show good reliability and validity for the assessment of study engagement among Japanese students (15). The internal consistency and test–retest reliability were Cronbach's α = 0.95 and *r* = 0.66 (*p* < 0.01), respectively. As for the criterion-related validity, there were significant correlations among the UEWS-S-J, social support, resilience, and subjective happiness.

### Mental Health

Participants were asked to evaluate the level of their current stress on a scale of 1 to 4, with 1 being a little stressed and 4 being very stressed. Fatigue, anxiety, and depression were each assessed as a binary variable, with 1 defined as yes and 0 as no.

### Lifestyle Habits

Participants were asked to report their total sleep time on both weekdays and holidays during the past month on the following scale: 1 = <5 h, 2 = 5–6 h, 3 = 6–7 h, 4 = 7–8 h, 5 = 8–9 h, and 6 = >9 h. These scores were replaced with approximate times: 4.5, 5.5, 6.5, 7.5, 8.5, and 9.5 h, respectively. Degree of restorative sleep in the past month was assessed on a scale of 1–4 (1 = very good; 4 = not good at all).

Regarding eating habits, the mean number of meals and snacks per day in the past month were assessed on the following scale: 1 = 0, 2 = 1, 3 = 2, 4 = 3, and 5 = ≥4. The score of 5 was replaced with 4 times per day in the statistical analysis.

### Statistical Analysis

Descriptive statistics were calculated for all variables. A two-step cluster analysis tested whether different groupings could be found based on the two indicators of study engagement and stress, which are important factors for maintaining better adaptation to college life (16, 17). Notably, the cluster analysis determined the number of clusters by the pattern-detection algorithm and not by the researcher's judgment. The Bayesian information criterion was calculated to identify the optimal quality of the clustering model. The two-step cluster analysis identified three clusters. Analysis of variance (ANOVA) with Welch adjustment, the Kruskal–Wallis test, and the chi-square test were employed to compare data among the three clusters. Effect size was calculated by η^2^ with ANOVA and Cramer's V with the chi-square test. Variables with a significant difference were subjected to *post-hoc* tests using the Games–Howell test, Dann–Bonferroni correction, and Bonferroni correction.

To examine differences in the proportion of participants with mental health problems between different groups based on cluster analysis, Cramer's V with chi-square test was used to compare the presence of fatigue, anxiety, and depression. Items with a significant difference were subjected to *pots-hoc* test using the Bonferroni correction.

To identify predictors of cluster membership, a logistic regression analysis was performed while controlling for potential confounders. The variables were category of cluster membership as a dependent variable. Two clusters with the highest and lowest mental health problems were regarded as the dependent variable. Sex (male vs. female), age, faculty (science vs. humanities), subjective benefits of online education (yes vs. no/not applicable), and lifestyle habit factors were the independent variables. Independent variables with the following two criteria were excluded to avoid multicollinearity: Pearson product-moment correlation coefficient among independent variables >0.70; and variance inflation factor >10. The sample size required for this logistic regression was over 90 participants in the smaller of the two outcome groups, in which the event per variable of 10 was calculated. The present study achieved this criterion.

All analyses were conducted using SPSS 27; a *p*-value of < 0.05 was considered statistically significant.

### Ethical Considerations

All procedures contributing to this work complied with the ethical standards of the relevant national and institutional committees on human experimentation and with the Helsinki Declaration of 1975, as revised in 2008. All procedures involving human subjects were approved by the ethics committee at the Institute for Datability Science of Osaka University (July 21, 2020).

## Results

### Participant Characteristics and Cluster Classifications

Table 1 shows the characteristics of the 1,259 students. Most participants were male (64.6%). The mean age of the sample was 18.67 years (.78), ranging from 18 to 24 years. Among them, 785 participants (62.5%) were enrolled in the science faculty and the remaining participants (37.5%) were part of the humanities faculty. Significant differences were observed among three clusters in terms of age, UWES-S-J score, stress, benefits of online education, sleep time on weekdays, restorative sleep, and number of meals, but not in terms of sex, faculty, sleep time on holidays, and number of snacks.

**Table 1:** Participant characteristics and cluster classifications.

**Variable**	**Whole sample *n* = 1,259**	**Cluster 1 *n* = 597**	**Cluster 2 *n* = 277**	**Cluster 3 *n* = 385**	***P***	**ES^b^**
**Sex, *n*(%)**						
Male	813 (64.6)	391 (65.5)	190 (68.6)	232 (60.3)	0.07^1)^	
Female	446 (35.4)	206 (34.5)	87 (31.4)	153 (39.7)		
Age, mean years (SD)	18.67 (0.78)	18.61 (0.77)	18.73 (0.80)	18.72 (0.76)	<0.05^2)^, A<C	
**Faculty, *n* (%)**						
Science	787 (62.5)	367 (61.5)	181 (65.3)	239 (62.1)	0.535^1)^	
Humanities	472 (37.5)	230 (38.5)	96 (34.7)	146 (37.9)		
UWES-S-J^a^, mean (SD)	46.17 (13.97)	53.34 (10.81)	29.62 (7.31)	49.10 (7.44)	<0.001^3)^ B<C<A	η^2^ = 0.570
Stress, mean (SD)	2.51 (0.86)	1.80 (0.41)	3.05 (0.81)	3.22 (0.42)	<0.001^2)^ A<B<C	
**Benefits of online education, *n* (%)**						
Yes	486 (38.6)	301 (50.4)	64 (23.1)	121 (31.4)	<0.001^1)^	Cramer's *V* = 0.224
No	363 (28.8)	96 (16.1)	138 (49.8)	129 (33.5)		
Not applicable	410 (32.6)	200 (33.5)	75 (27.1)	135 (35.1)		
**Sleep, mean (SD)**						
Sleep time on weekdays	6.80 (0.98)	6.89 (0.94)	6.77 (1.12)	6.66 (0.91)	<0.01^3)^C<A	η^2^ = 0.011
Sleep time on holidays	7.42 (1.01)	7.42 (1.01)	7.49 (1.19)	7.38 (1.08)	0.468^3)^	–
Restorative sleep	1.86 (0.71)	1.67 (0.63)	2.03 (0.82)	2.04 (0.69)	<0.001^2)^	
**Food, mean (SD)**						
Number of meals	2.71(0.55)	2.74 (0.52)	2.64 (0.60)	2.71 (0.58)	<0.05^2)^	
Number of snacks	0.89 (0.84)	0.87 (0.79)	0.95 (0.97)	0.89 (0.83)	B < A 0.940^2)^	

a *UWES-S-J, Japanese version of the Utrecht Work Engagement Scale for Students;*

b *ES, effect size.*

*A, cluster 1; B, cluster 2; C, cluster 3.*

1) *Cramer's V with chi-square.*

2) *Kruskal–Wallis test, post-hoc test = Bonferroni correction.*

3) *ANOVA with Welch adjustment, post-hoc test = Games–Howell test.*

**Figure 2:**
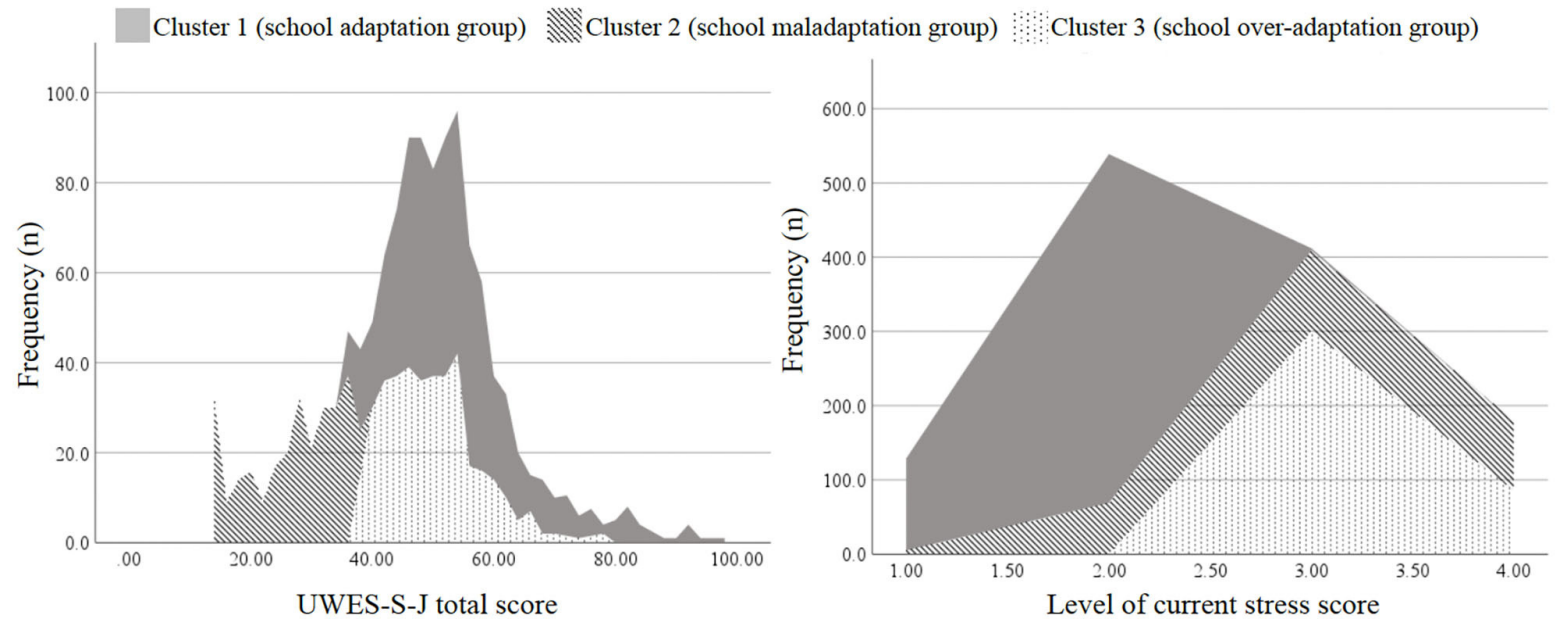
Score distribution of the three clusters in UWES-S-J and current stress. UWES-S-J, Japanese version of the Utrecht Work Engagement Scale for Students. The left figure shows the distribution of the UWES-S-J total score among three clusters. The right figure shows the distribution of the level of current stress score among the three clusters.

Two-step cluster analysis based on the UWES-S-J score and the level of current stress determined three clusters. Figure 2 shows the distribution of each score among the clusters. The first cluster (school adaptation group) had the highest UWES-S-J scores and lowest levels of current stress. The second cluster (school maladaptation group) showed the lowest UWES-S-J scores and somewhat high levels of current stress. The third cluster (school over-adaptation group) had somewhat high UWES-S-J scores and the highest levels of current stress. Significant differences existed among the three groups in terms of age, subjective benefits of online education, total sleep time on weekdays, degree of restorative sleep, and mean number of meals per day (Table 1). The effect size was small to medium.

### Mental Health Problems

**Figure 3:**
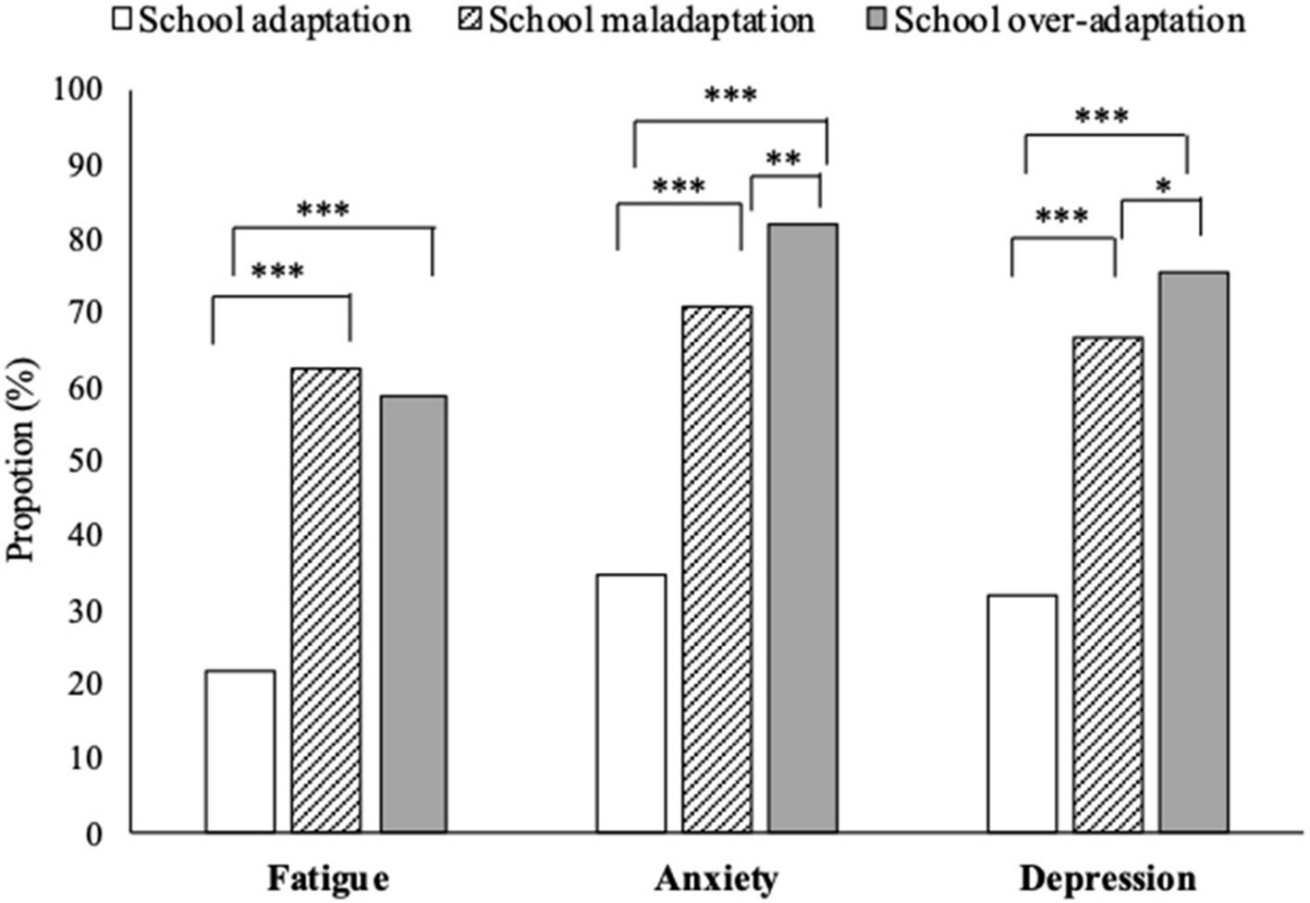
Proportion of mental health problems according to the three clusters. Chi-square test with Bonferroni correction ***p < 0.001, **p < 0.01, *p < 0.05. Participants in the school over-adaptation group significantly had the most anxiety and depression among the three groups. Participants in the school maladaptation and over-adaptation groups had significantly more fatigue than those in the school adaptation group. The effect size regarding the comparison of fatigue, anxiety, and depression was medium to large (Cramer's V = 0.392, 0.436, and 0.408, respectively).

Figure 3 compares the proportion of participants with mental health problems.

Participants in cluster 3 (school over-adaptation group) significantly evinced the most mental health problems, except for fatigue. Those in cluster 1 (school adaptation group) had the lowest mental health problems. Participants in Cluster 2 (school maladaptation group) had significantly more fatigue, anxiety, and depression problems than those in cluster 1. The effect size for the comparison of fatigue, anxiety, and depression was medium to large (Cramer's *V* = 0.392, 0.436, and 0.408, respectively).

### Logistic Regression Analysis

Table 2 demonstrates the logistic regression results. The dependent variable is membership of the school over-adaptation group with reference to the school adaptation group. The overall model explained 67.6% of the variance. The model's goodness of fit was acceptable (Hosmer–Lemeshow test, *p*-value = 0.946). Sex, age, subjective benefits of online education, total sleep time on weekdays and on holidays, and degree of restorative sleep were significant predictors of cluster membership. Students who were female, older, considered online education to be less beneficial, had shorter total sleep time on weekdays, had longer total sleep time on holidays, and had worse restorative sleep were more likely to be classified into the school over-adaptation group.

**Table 2:** Logistic regression analysis predicting school over-adaptation.

**Variables**	**B**	**Odds ratio**	**95% confidence interval**		***p***
			**Lower**	**Upper**	
**Sex**						
Male		1.00 (ref)			<0.05
Female	0.352	1.42	1.06	1.91	
Age	0.187	1.21	1.01	1.44	<0.05
**Faculty**						
Science	0.046	1.05	0.781	1.40	0.760
Humanities		1.00 (ref)			
**Benefits of online education**						
Yes		1.00 (ref)			<0.001
No/Not applicable	0.777	2.17	1.64	2.88	
Sleep time on weekdays	−0.192	0.826	0.683	0.998	<0.05
Sleep time on holidays	0.192	1.21	1.03	1.43	<0.05
Restorative sleep	0.821	2.27	1.81	2.86	<0.001
Number of meals	−0.019	0.981	0.760	1.27	0.884
Number of snacks	−0.075	0.927	0.781	1.10	0.390

*Logistic regression analysis with cluster memberships of Cluster 3 (school over-adaptation group) as a dependent variable and sex (male vs. female), age, faculty (science vs. humanities), subjective benefits of online education (yes vs. no/not applicable), sleep time on weekdays, sleep time on holidays, restorative sleep, number of meals, and number of snacks as independent variables*

## Discussion

This study clarified characteristics of school adaptation in University students exposed to the drastic transition in the educational system from traditional face-to-face classes to fully online classes during the COVID-19 pandemic, while also exploring the accompanying mental health problems and factors associated with school adaptation. Two major findings emerged. First, after the participants were statistically classified into three groups based on study engagement and stress in the two-step cluster analysis (school adaptation, school maladaptation, and school over-adaptation), the school over-adaptation group was found to have the most mental health problems among the three groups. Second, participants who were female, older, considered online education to be less beneficial, and had shorter total sleep time on weekdays, longer total sleep time on holidays, and worse restorative sleep were more likely to belong to the school over-adaptation group.

The two-step cluster analysis statistically linked the three school adaptation groups according to study engagement and stress. This implied that some students who suddenly experienced fully online education superficially adapted to the new school environment while exhibiting many mental health problems. The present results differ from a previous study that found three patterns of school success in a period with the usual education system: well-adjusted average academic achiever; average-adjusted high achiever, and low-adjusted low achiever (18), although it is difficult to compare the findings directly owing to methodological differences. Difficulty in maintaining motivation could explain the characteristics of school adaptation in students exposed to online education. A previous study (4) examined challenges to online education, in which some students positively preferred online education, while others considered it unacceptable, suggesting that not all students could adapt to the rapid transition to a new academic environment. The present results may support the findings of Terenko and Ogienko (19), who showed that a significant concern with administering online learning was maintaining students' motivation, an important factor for school adaptation. Moreover, during the COVID-19 pandemic, students' motivation was more likely to be affected by their living environment, economic conditions, or lack of psychosocial activities, such as communication with friends or extracurricular activities, and the transition of the education mode (20, 21). Indeed, this study found that students in the school maladaptation and over-adaptation groups presented more mental health problems, such as fatigue, anxiety, and depression, although the causes of these mental health problems could not be clearly determined. This finding is in line with several other studies that have demonstrated psychological stress and low well-being in University students during the COVID-19 pandemic (22, 23). Academic staff should particularly recognize the presence of a school over-adaptation cluster whose members can barely maintain high study engagement while exhibiting high levels of stress during rapid transitions.

Participants who were female, older, experienced poor subjective benefits of online education, and had shorter sleep time on weekdays, longer sleep time on holidays, and worse restorative sleep were more likely to be in the school over-adaptation group, based on the logistic regression analysis. To the best of our knowledge, limited data exist regarding the predictors of school over-adaptation for students undergoing fully online education during the COVID-19 pandemic. An interesting new finding is that students who did not recognize the advantages of online education were more likely to be classified in the school over-adaptation group. Thus, academic staff should consider how to utilize online classes with their lectures to improve students' ratings of subjective benefits of online education. The result concerning sleep problems is partly in line with other studies that have demonstrated an association between sleep difficulties and stress or mental health problems in University students, although these were not particular to the COVID-19 pandemic (24, 25). Concerning demographic variables associated with school adaptation, this study found that female and older students were significantly more likely to experience school over-adaptation during the COVID-19 pandemic. The association of sex and age with school adaptation among University students has been a controversial issue (17, 26–28). Cabras and Mondo (29) indicated that sex could play an important role in building a person's coping strategy. In general, late adolescent girls are more likely than boys to use emotion-focused coping strategies (30), which may require mutual face-to-face interaction with others or enrichment of leisure activities. Such emotion-focused coping strategies that female students prefer to use might be restricted during the COVID-19 pandemic due to social distancing or stay-at-home requirements. This restriction of coping strategies may explain why female students had higher odds of over-adaptation for inexperienced stress in the sudden transition of the education system from face-to-face teaching to the online mode. This explanation is partially supported by a previous study which found a more pronounced negative effect of the COVID-19 pandemic on female students' academics, social isolation, stress, and mental health compared to male students (11). Regarding the effect of age on school adaptation, the present study's finding differs from several previous studies which showed that older University students tended to have a better perception of online education than younger students (31, 32), although the outcome of adaptation for the rapid transition in the education system in the present study is not comparable to those studies. This difference might be explained by the range in the level of education targeted in each survey. The previous studies focused on students in different years of undergraduate study, while the present study included only those in the first year. Detailed research is needed to address these differences in sex and age in school adaptation of University students during the COVID-19 pandemic.

This study has several limitations. First, the cross-sectional design precludes comparison of students' temporal changes with the transition from face-to-face to online programs. Pre-existing mental health problems have been predicted to affect students' current outcomes during the COVID-19 pandemic (33). Social support from family or friends could also affect University students' mental health problems (34). Therefore, longitudinal methods should be employed to further address temporal changes in their mental health. Second, this study did not include other factors, such as changes in participants' economic situation, interactions with friends, or daily living activities, which would have been disturbed by the COVID-19 pandemic. This limitation suggests the need for further investigation to understand several potential factors affecting the high stress levels of students in the school over-adaptation and maladaptation groups. Third, the study lacked robust and validated measures for stress, depressive symptoms, anxiety, sleep, and eating habits to evaluate the underlying associations accurately. Although this study comprehensively assessed various issues associated with University students' life, by considering a large sample and including factors such as mental health problems or lifestyle habits, the lack of validated measures could affect data accuracy. Fourth, this study included only students in the first year of undergraduate study, who provided complete assessment data, from a single University without a comparison group, although Osaka University is one of the largest national universities in Japan and has 11 undergraduate and 16 graduate schools. The finding of this study may not be generalizable for postgraduate students or the entire undergraduate student population in Japan. Collectively, a prospective study to comprehensively investigate the potential effects of these factors on students' engagement is needed. Despite these limitations, an important strength was that the methodology employed in this study enabled an exploratory investigation of school adaptation to the rapid transition of the education system in a large sample of first year undergraduate students using an online questionnaire. Additionally, the present study focused on undergraduate students in terms of both mental health and online education, while many previous studies sought to indirectly address the issues associated with each of the domains.

This is the first study to reveal the characteristics of school adaptation in University students exposed to a fully online education system during the COVID-19 pandemic, and to identify the mental health characteristics and factors associated with school adaptation.

The results suggest that academic staff members should provide extensive counseling to support students' mental health to prevent overlooking the existence of school over-adaptation and its risk factors, although providing online programs requires substantial resources. Future studies should identify temporal changes in students' mental health using comprehensive assessment tools. Students' perception of online education would play an important role in examining the effect of the rapid transition from traditional face-to-face teaching to online education.

## Data Availability Statement

The datasets generated during the current study are not publicly available due to privacy considerations of the participants. Requests to access the datasets should be directed to Hiroyoshi Adachi, hadachi@psy.med.osaka-u.ac.jp.

## Ethics Statement

The studies involving human participants were reviewed and approved by the ethics committee at the Institute for Datability Science of Osaka University. The patients/participants provided their written informed consent to participate in this study.

## Author Contributions

HA, HN, SS, and HT conceived the design and concept of this study. DI, HA, SS, and HT analyzed the data and DI wrote the first draft. NT, AM, TH, YY, and MI advised about the data analysis and interpretation. All authors contributed to the article and approved the submitted version.

## Funding

This work was partially supported by Innovation Platform for Society 5.0 of the Japan Ministry of Education, Culture, Sports, Science and Technology (Code: S004541). The funder of the study had no role in data collection, data analysis, data interpretation, writing of the report, or decision to submit the paper for publication.

## Conflict of Interest

The authors declare that the research was conducted in the absence of any commercial or financial relationships that could be construed as a potential conflict of interest.

## Publisher's Note

All claims expressed in this article are solely those of the authors and do not necessarily represent those of their affiliated organizations, or those of the publisher, the editors and the reviewers. Any product that may be evaluated in this article, or claim that may be made by its manufacturer, is not guaranteed or endorsed by the publisher.
