# Supplementary material for: Characteristics of Adaptation in Undergraduate University Students Suddenly Exposed to Fully Online Education During the COVID-19 Pandemic
Source: Front Psychiatry. 2021 Sep 13;12:731137. doi: 10.3389/fpsyt.2021.731137 (PMC8473868; doi:10.3389/fpsyt.2021.731137)
Supplement: Supplementary file 1 [file Data_Sheet_1.docx]

**Survey for Evaluating Effects of online classes on the Academic, Psychological, and Living Conditions**

***These questions are related to online classes attended.***

1. What format of online classes (excluding seminars and practical) have you attended? (Note: multiple selections are permitted):

I. Simultaneous bidirectional real-time lecture distribution format

II. Simultaneous bidirectional real-time meeting format

III. On-demand (internet distribution method) lecture video distribution format

IV. On-demand (internet distribution method) resource/coursework distribution format

V. Other

2. Was the “simultaneous bidirectional real-time lecture distribution format” online classes (excluding seminars and practical) that you have attended, easy to attend?

I. Agree, II. Slightly agree, III. Neutral, IV. Slightly disagree, V. Disagree, VI. Have not attended one

1. Was the “simultaneous bidirectional real-time lecture meeting format” online classes (excluding seminars and practical) that you have attended, easy to attend?

I. Agree, II. Slightly agree, III. Neutral, IV. Slightly disagree, V. Disagree, VI. Have not attended one

1. Was the “on-demand (internet distribution method) lecture video distribution method” online classes (excluding seminars and practical) that you have attended, easy to attend?

I. Agree, II. Slightly agree, III. Neutral, IV. Slightly disagree, V. Disagree, VI. Have not attended one

1. Was the “on-demand (internet distribution method) resource/coursework distribution method” online classes (excluding seminars and practical) that you have attended, easy to attend?

I. Agree, II. Slightly agree, III. Neutral, IV. Slightly disagree, V. Disagree, VI. Have not attended one

1. Compared to regular face to face classes, did you experience any advantages of these online classes？

I. Agree, II. Slightly agree, III. Neither, IV. Slightly disagree, V. Disagree, VI. Have not attended one

1. Currently, are you experiencing any issues with regard to attending online classes? (Note: multiple responses are permitted)

・It is difficult to maintain motivation

・The amount of coursework is high

・It is physically exhausting

・It is difficult to speak up during classes

・It is difficult to ask the teacher questions

・I am hesitant to show my face on video chats

・I cannot keep up with the classes

・My life rhythm gets disrupted

・I am worried about whether I can get credits

・There are no issues in particular

・Other

***What is your current academic (online classes) approach?***

1. When I’m studying, I feel mentally strong.

0.Never, I.Almost never, II.Rarely, III.Sometimes, IV.Often, V.Very Often, VI.Always

1. I can continue for a very long time when I am studying.

0.Never, I.Almost never, II.Rarely, III.Sometimes, IV.Often, V.Very Often, VI.Always

1. When I study, I feel like I am bursting with energy

0.Never, I.Almost never, II.Rarely, III.Sometimes, IV.Often, V.Very Often, VI.Always

1. When studying (online classes), I feel strong and vigorous.

0.Never, I.Almost never, II.Rarely, III.Sometimes, IV.Often, V.Very Often, VI.Always

1. When I get up in the morning, I feel like going to online class.

0.Never, I.Almost never, II.Rarely, III.Sometimes, IV.Often, V.Very Often, VI.Always

1. I find my studies to be full of meaning and purpose.

0.Never, I.Almost never, II.Rarely, III.Sometimes, IV.Often, V.Very Often, VI.Always

1. My studies inspire me

0.Never, I.Almost never, II.Rarely, III.Sometimes, IV.Often, V.Very Often, VI.Always

1. I am enthusiastic about my studies.

0.Never, I.Almost never, II.Rarely, III.Sometimes, IV.Often, V.Very Often, VI.Always

1. I am proud of my studies

0.Never, I.Almost never, II.Rarely, III.Sometimes, IV.Often, V.Very Often, VI.Always

1. I find my studies challenging

0.Never, I.Almost never, II.Rarely, III.Sometimes, IV.Often, V.Very Often, VI.Always

1. Time flies when I’m studying

0.Never, I.Almost never, II.Rarely, III.Sometimes, IV.Often, V.Very Often, VI.Always

1. When I am studying, I forget everything else around me

0.Never, I.Almost never, II.Rarely, III.Sometimes, IV.Often, V.Very Often, VI.Always

1. I feel happy when I am studying intensively

0.Never, I.Almost never, II.Rarely, III.Sometimes, IV.Often, V.Very Often, VI.Always

1. I can get carried away by my studies.)

0.Never, I.Almost never, II.Rarely, III.Sometimes, IV.Often, V.Very Often, VI.Always

***What is your current psychological state?***

1. Do you feel stress?

I. Almost never, II.Sometimes, III.Frequently, IV.All the time

1. Do you feel tired?

I.Yes, II.No

1. Do you feel anxious?

I.Yes, II.No

1. Do you feel depressed?

I.Yes, II.No

***What have been your lifestyle habits over the past month?***

***(i.e., upon beginning to partake in online classes)***

1. What time do you go to sleep on the weekdays?

I. By 10PM, II.10PM-12AM, III.12-2AM, IV.2-4AM, V.4-6AM, VI.6AM onward

1. What time do you wake up on the weekdays?

I.Before 4 AM, II.4-6AM, III.6-8 AM, IV.8-10 AM, V.10AM-12PM, VI.12PM onward

1. What are your sleeping hours on the weekday?

I. Less than 5 hours, II.5-6 hours, III.6-7 hours, IV.7-8 hours, V.8-9 hours, VI.9 hours or more

1. What is your sleeping time on weekends/holidays?

I. By 10PM, II.10PM-12AM, III.12-2AM, IV.2-4AM, V.4-6AM, VI.6AM onward

1. What time do you wake up on weekends/holidays?

I.Before 4 AM, II.4-6AM, III.6-8 AM, IV.8-10 AM, V.10AM-12PM, VI.12PM onward

1. What are your sleeping hours on weekends/holidays?

I. Less than 5 hours, II.5-6 hours, III.6-7 hours, IV.7-8 hours, V.8-9 hours, VI.9 hours or more

1. Do you believe that you get sufficient rest with your sleep?

I.A lot, II.Somewhat, III.Not much, IV.Never

1. How many meals do you eat a day?

I.There are days when I do not eat, II.Once, III.Twice, IV.Three times, V.Four or more times

1. How many times a day do you feel as though you have no appetite?

I.0 times, II.Once, III.Twice, IV.Three times, V.I never have an appetite

1. How many times a day do you eat snacks?

I.I do not have a habit of eating snacks, II.Once, III.Twice, IV.Three times, V.Four or more times
